# Microbial Dysbiosis, Infection, and Immunometabolic Signaling in Polyendocrine Metabolic Ovarian Syndrome (Formerly PCOS)

**DOI:** 10.3390/pathogens15101003

**Published:** 2026-09-22

**Authors:** Allyson Nevins, Nicholas Stansbury, Randal D. Robinson, Guangming Zhong

**Affiliations:** 1 Department of Obstetrics and Gynecology, UT Health San Antonio, San Antonio, TX 78229, USA; nevinsa1@uthscsa.edu (A.N.); stansburyn@uthscsa.edu (N.S.); robinsonr3@uthscsa.edu (R.D.R.); 2 Department of Microbiology, Immunology and Molecular Genetics, UT Health San Antonio, San Antonio, TX 78229, USA

**Keywords:** polyendocrine metabolic ovarian syndrome, polycystic ovary syndrome, gut microbiome, vaginal microbiome, dysbiosis, inflammation, microbial infection, bile acids, interleukin-22, STAT4

## Abstract

Polyendocrine metabolic ovarian syndrome (PMOS), recently proposed as a new name for polycystic ovary syndrome (PCOS), is a heterogeneous endocrine–metabolic disorder characterized by hyperandrogenism, ovulatory dysfunction, and variable ovarian morphology. Low-grade inflammation is common, but its initiating and sustaining factors differ among phenotypes. Microbial communities have emerged as potential modifiers of this inflammatory and metabolic environment. Human studies generally identify differences in gut microbial community structure between affected and unaffected women, although no universal taxonomic signature has been reproduced across regions, phenotypes, or analytic platforms. Mechanistic studies implicate altered intestinal barrier signaling, microbial metabolites, bile acid-interleukin-22 pathways, and immune-cell polarization; however, several influential pathways remain supported principally by animal models. Evidence for lower genital tract dysbiosis is more limited, and apparent associations may reflect hormonal state, menstrual irregularity, adiposity, medications, sexual exposures, or sampling methods. Evidence that specific infections cause PMOS/PCOS is sparse and inconsistent. Small serologic studies involving *Chlamydia* species and *Helicobacter pylori* do not establish temporality or causality. This review distinguishes dysbiosis from infection, evaluates human and experimental evidence, and proposes a bidirectional microbial–immune–metabolic–ovarian framework. A focused hypothesis involving interleukin-12-STAT4 signaling is also examined because it provides a specific, testable framework linking microbial or sterile inflammatory signaling to ovarian dysfunction. STAT4 can promote inflammatory T-helper-1 programs and granulosa-cell apoptosis in non-PCOS models, while estrogen can enhance STAT4 activation; nevertheless, direct human PMOS/PCOS evidence is currently absent, and the proposed pathway should not be interpreted as an established disease mechanism. Future studies require deep phenotyping, longitudinal multi-omics, compartment-specific sampling, standardized analysis, and mechanistic validation before microbiome-directed or immune-directed interventions can enter clinical care.

## 1. Introduction

In 2026, an international multistakeholder consensus process proposed polyendocrine metabolic ovarian syndrome (PMOS) as a new name for polycystic ovary syndrome (PCOS), with the aim of replacing anatomically misleading terminology and better reflecting the condition’s endocrine, metabolic, and ovarian manifestations [1]. The terminology is undergoing a period of transition and has not yet been universally incorporated into professional-society recommendations or clinical guidelines. Nevertheless, several professional organizations have endorsed the change, including the American Society for Reproductive Medicine (ASRM) [2]. Accordingly, this review uses PMOS when referring to the contemporary disease concept while retaining “PCOS” when describing previously published cohorts, experimental models, diagnostic criteria, or literature indexed under the established term.

PMOS/PCOS affects approximately one in eight women, although prevalence varies with diagnostic criteria, age, ethnicity, and ascertainment [1,3,4]. Current diagnostic frameworks require combinations of ovulatory dysfunction, clinical or biochemical hyperandrogenism, and polycystic ovarian morphology or anti-Müllerian hormone-based criteria in appropriately selected adults after exclusion of mimicking disorders [3,4]. The syndrome encompasses reproductive, metabolic, dermatologic, and psychological manifestations and cannot be reduced to ovarian morphology. Insulin resistance may occur independently of body mass index, but adiposity frequently amplifies dysglycemia, hyperandrogenism, and inflammatory signaling [4,5,6].

Low-grade systemic inflammation is reproducibly associated with PCOS, yet interpretation is difficult because adiposity, insulin resistance, sleep disorders, diet, and medications are themselves inflammatory modifiers [7]. Importantly, inflammation is not a unitary cause. It may arise from adipose tissue, altered nutrient signaling, oxidative and endoplasmic reticulum stress, ovarian immune-cell activation, epithelial barrier dysfunction, microbial products, or infection. These processes can form reinforcing loops: hyperandrogenism and insulin resistance can alter mucosal environments, while microbial products and metabolites can modify endocrine and immune signaling.

The original “dysbiosis of gut microbiota” hypothesis proposed that increased intestinal permeability and translocation of bacterial products could promote insulin resistance and ovarian androgen excess [8]. Subsequent human cohorts, reanalyses, animal transfer experiments, and metabolomic studies have expanded that concept but have also exposed its limitations. The central question is no longer whether the microbiome differs in PCOS; rather, it is which microbial functions are reproducible, whether they precede disease, which host phenotypes they modify, and whether changing them improves clinically meaningful outcomes.

This narrative review has four objectives: (1) critically evaluate gut and female genital tract microbiome findings; (2) distinguish microbial dysbiosis from infection; (3) integrate microbial signals with systemic and intraovarian inflammation; and (4) define testable mechanistic hypotheses, including a circumscribed role for IL-12-STAT4 signaling. The review emphasizes evidence strength and translational limitations rather than presenting microbial associations as established causes.

## 2. Literature Identification and Interpretive Framework

This narrative review used a targeted literature search to identify representative clinical and mechanistic evidence rather than to exhaustively enumerate all eligible records. PubMed/MEDLINE was searched from database inception through 8 August 2026. Searches combined “polycystic ovary syndrome” or “polyendocrine metabolic ovarian syndrome” with terms addressing four domains: (1) gut microbial ecology and function (“gut microbiome,” “gut microbiota,” “dysbiosis,” “intestinal permeability,” “lipopolysaccharide,” “bile acid,” “short-chain fatty acid,” and “IL-22”); (2) the female genital tract and infection (“vaginal microbiome,” “female genital tract,” “microbial infection,” “*Chlamydia*,” and “*Helicobacter pylori*”); (3) inflammatory and immune pathways (“inflammation,” “inflammasome,” “cGAS-STING,” “IL-12,” “STAT4,” “T helper 1,” “regulatory T cell,” and “granulosa cell”); and (4) experimental models and interventions (“animal model,” “fecal microbiota transplantation,” “probiotic,” and “synbiotic”). Terms within and across domains were combined as appropriate using the Boolean operators AND and OR. Backward and forward citation chaining from key reviews, guidelines, and primary studies was used to identify additional relevant publications.

Eligible sources were English-language, peer-reviewed publications that directly informed the review objectives. These included human observational studies and clinical trials; systematic, meta-analytic, and scoping reviews; international guidelines; and experimental animal or in vitro studies providing mechanistic evidence relevant to microbial ecology, infection, inflammation, metabolic signaling, or ovarian function in PMOS/PCOS. Older studies were retained when they established a widely used hypothesis, pathway, or experimental model. Preprints, conference abstracts, editorials, non-peer-reviewed material, duplicate reports, publications without sufficient methodological or outcome information, and studies without direct relevance to the prespecified review domains were excluded.

One author screened titles and abstracts for relevance and reviewed the full text of potentially eligible publications. A second author reviewed the selected literature to confirm eligibility, the accuracy of extracted factual claims, and balanced representation of study strengths, limitations, and potential sources of bias. Differences were resolved through discussion. Evidence was interpreted according to study design: human cross-sectional studies were considered associative and vulnerable to reverse causation and confounding; longitudinal human studies could support temporal sequence but remained observational; transfer, depletion, supplementation, rescue, or genetic-perturbation experiments could support causality within a model but not necessarily in humans; and randomized trials were required to establish clinical efficacy. Mendelian-randomization analyses were treated as hypothesis-generating because microbial instruments are often weak, taxonomically coarse, and derived from populations that differ from PCOS cohorts. Because this was a narrative review, no systematic-review protocol, formal risk-of-bias instrument, quantitative synthesis, or claim of exhaustive study identification was undertaken.

## 3. Inflammation in PMOS/PCOS: Systemic Signal and Ovarian Context

Meta-analytic evidence supports higher circulating C-reactive protein and selected inflammatory mediators in women with PCOS, including after statistical adjustment for adiposity in some analyses [7]. However, residual confounding remains substantial, and cytokine concentrations vary with assay, phenotype, menstrual status, medication, and metabolic disease. It is therefore more accurate to describe inflammation as a common disease amplifier than as a universal initiating event.

The ovary provides a plausible site where systemic metabolic stress becomes a reproductive phenotype. Human granulosa-lutein cells from women with PCOS show enrichment in inflammatory transcripts, cytokines, chemokines, and immune cell markers; androgen and gonadotropin exposure can further increase cytokine production [9]. Follicular fluid studies also suggest that peripheral inflammatory signals can activate granulosa cell inflammatory programs [10]. Hyperandrogenic experimental environments can polarize macrophages toward proinflammatory states that impair granulosa-cell function [11]. These findings support a local feed-forward circuit involving androgens, theca and granulosa cells, and resident or recruited immune cells.

Microbial signals could enter this circuit through at least three routes. First, intestinal or genital epithelial disruption may increase exposure to lipopolysaccharide (LPS), peptidoglycan fragments, or microbial nucleic acids. Second, microbial metabolites—including secondary bile acids, short-chain fatty acids (SCFAs), and tryptophan products—may alter immune, hepatic, adipose, or ovarian signaling. Third, infection may induce episodic or sustained inflammation. These routes should not be conflated: dysbiosis is a change in community structure or function, whereas infection implies invasion or pathogenic replication and requires organism-specific evidence.

## 4. Human Gut Microbiome Evidence

### 4.1. Community Diversity and Composition

Early PCOS studies reported lower alpha diversity and differences in taxa such as *Bacteroides*, *Prevotella*, *Escherichia-Shigella*, *Akkermansia*, and *Lactobacillus* [12,13,14]. These observations generated valuable hypotheses but did not yield a reproducible diagnostic signature. A 2024 individual-level reanalysis of 14 publicly available studies and 948 participants found no significant overall difference in alpha diversity, despite differences in community composition [15]. Signals varied by geography and testosterone phenotype: *Alistipes* was enriched predominantly in European cohorts, while *Blautia* and *Roseburia* were more abundant in Chinese cohorts; women with higher testosterone also differed from those with lower testosterone [15]. This heterogeneity argues against describing PCOS as a single microbial state.

Recent systematic reviews similarly conclude that beta-diversity and functional differences are more consistent than any single taxon and that human and animal findings often diverge [16,17,18]. Relevant sources of heterogeneity include diagnostic criteria, phenotype distribution, body composition, diet, age, ethnicity, geography, stool collection, DNA extraction, variable regions of the 16S rRNA gene, sequencing depth, taxonomic database, batch correction, and medication exposure. Metformin, hormonal contraceptives, antibiotics, anti-obesity drugs, probiotics, and dietary changes can each alter microbiome measurements. Many studies are too small to control these factors.

These factors also complicate interpretation because they are biologically related to both PCOS and the microbiome. Obesity and insulin resistance can independently alter microbial ecology and systemic inflammation; diet supplies microbial substrates; and metformin, antibiotics, anti-obesity therapies, probiotics, and hormonal contraception can change microbial composition or function. Menstrual phase, irregular bleeding, and exogenous hormone exposure may further affect sampling conditions and sex-steroid-dependent microbial niches. Some studies report case–control differences after adjustment for selected variables such as age, body mass index, or metabolic measures, but covariate sets are inconsistent, and few studies simultaneously account for adiposity, directly measured insulin resistance, habitual diet, medication exposure, hormonal contraception, and menstrual status [15,16,17,18]. Accordingly, current evidence does not establish that reported microbiome differences persist after comprehensive adjustment, and residual confounding remains a major limitation.

The most defensible human conclusion is therefore narrow: PCOS is associated with altered gut community structure in many cohorts, but the direction and identity of taxonomic changes are context-dependent. Functional pathways and metabolites may prove more informative than genus-level abundance.

### 4.2. Intestinal Barrier Function and Endotoxemia

The intestinal-barrier hypothesis proposes that dysbiosis increases permeability, allowing LPS or other microbial products to reach the circulation and activate TLR4-NF-κB and inflammasome pathways. In obesity models, metabolic endotoxemia can promote inflammation and insulin resistance [19]. Human PCOS evidence is mixed. A pilot study found altered stool microbial composition but did not identify gut barrier dysfunction or endotoxemia as group-level drivers [12]. In contrast, a later cohort reported elevations in LPS and LPS-binding protein, and experimental work showed that inflammatory stimuli can increase theca-interstitial androgen production [20,21]. A six-month synbiotic trial reported reductions in LPS-related markers alongside metabolic and androgen changes, although nearly half of participants discontinued and gut microbial composition was not measured [22].

These results support barrier dysfunction as a plausible mechanism in a subset, not a defining feature of all PCOS. Direct permeability testing, validated LPS assays, diet and medication control, and paired stool/metabolite measurements are needed. LPS concentrations are particularly susceptible to preanalytical contamination and assay variability.

### 4.3. Microbial Metabolites

SCFAs are produced through fermentation of dietary substrates and regulate colonocyte energy, epithelial integrity, and immune differentiation. Butyrate and propionate can promote regulatory T-cell programs in experimental systems [23,24]. In PCOS, reported SCFA patterns are inconsistent, partly because fecal abundance does not directly measure production, absorption, or tissue exposure. A taxon capable of producing butyrate may not do so under a given dietary environment, and fecal concentration may rise because absorption is reduced. Functional metagenomics and isotope-informed metabolomics are therefore preferable to inferring function from taxonomy alone.

Bile-acid signaling provides the most developed experimental microbiome-to-ovary mechanism. In the human component of the study, Qi and colleagues reported enrichment of *Bacteroides vulgatus* and reduced circulating glycodeoxycholic acid and tauroursodeoxycholic acid in women with PCOS [25]. These human data establish associations but do not demonstrate that the bile-acid–IL-22 pathway causes PCOS. The directionality and rescue evidence came from mouse experiments: transfer of *B. vulgatus* or microbiota from women with PCOS produced reproductive and metabolic abnormalities, whereas administration of selected bile acids or IL-22 improved phenotypes; knockout experiments further supported a TGR5–IL-22 pathway within the model [25]. These experiments provide important insight into potential microbiome–immune–ovarian pathophysiology, but mouse models do not fully reproduce the heterogeneous human syndrome and cannot establish that the same mechanism or treatment effect occurs in humans. Thus, this remains an influential translational framework requiring independent human replication.

Tryptophan-derived indoles and aryl hydrocarbon receptor signaling are increasingly implicated in mucosal immunity and female reproductive physiology, but direct PCOS data remain less mature than the bile-acid literature [16,17]. Future studies should measure microbial genes and circulating or tissue-level metabolites together rather than treating predicted pathway abundance as equivalent to biochemical function.

### 4.4. Reverse Causation and the Androgen–Microbiome Loop

Hyperandrogenism may itself reshape the microbiome. Sex hormones alter intestinal motility, bile-acid metabolism, immune tone, and substrate availability, while insulin resistance and adiposity change diet and medication exposure. In humans, the association between microbial structure and testosterone phenotype in pooled data is compatible with both microbial effects on endocrine function and endocrine effects on microbes [15]. Animal studies showing that androgen exposure alters microbial communities provide mechanistic support for bidirectionality within those models, but they neither establish which direction dominates nor confirm the same causal relationship in humans.

Accordingly, future cohorts should collect repeated samples before and after endocrine transitions or treatment, including puberty, weight change, combined hormonal contraception, metformin, GLP-1 receptor agonists, ovulation induction, and pregnancy. A baseline case–control comparison cannot resolve temporal direction. The evidence supporting the role of human gut microbiome in PCOS is summarized in Table 1.

## 5. Female Genital Tract Microbiome

### 5.1. Hormonal Ecology and Mucosal Inflammation

In many reproductive-aged women, vaginal communities are dominated by one or more *Lactobacillus* species that maintain low pH through lactic-acid production. Other healthy individuals have stable, higher-diversity communities; therefore, “diversity” cannot be interpreted identically in the gut and vagina. In the gut, diversity is often treated as favorable, whereas in the vagina a highly diverse anaerobic community can be associated with bacterial vaginosis and mucosal inflammation. Vaginal community composition changes with estrogen, glycogen availability, menstrual bleeding, sexual exposures, pregnancy, contraceptives, and antibiotics [26,27].

*Lactobacillus*-deficient communities can be associated with higher concentrations of IL-1 family cytokines and chemokines, epithelial-barrier changes, and susceptibility to selected sexually transmitted infections [28,29,30]. Those observations establish general mucosal biology, not a PCOS-specific pathway. In PCOS, irregular bleeding, altered estrogen and progesterone exposure, hyperandrogenism, insulin resistance, obesity, and medication use could each change the genital environment. Thus, genital dysbiosis may be a consequence, modifier, or correlate of the syndrome.

The hormonal milieu of PMOS/PCOS may also differ qualitatively from normal cyclic variation. Sustained hyperandrogenemia and the reduced cyclic progesterone exposure associated with oligo-anovulation could influence epithelial maturation, glycogen availability, mucosal immunity, or vaginal pH in patterns that are not captured by a single estradiol measurement [3,4,26,27]. However, whether hyperandrogenemia per se, independent of menstrual irregularity, adiposity, insulin resistance, medication use, or exogenous hormones, alters vaginal community composition or function remains entirely unknown and represents a priority for future investigation. Studies addressing this gap should include repeated cycle- or bleeding-anchored sampling, biochemical androgen and progesterone measurements, and comparisons stratified by ovulatory status while controlling for adiposity, metabolic phenotype, hormonal contraception, and antimicrobial exposure.

### 5.2. PCOS-Specific Evidence

The PCOS genital-microbiome literature is small. A 2024 scoping review identified only five eligible studies and concluded that PCOS was associated with vaginal dysbiosis, but emphasized heterogeneity and insufficient evidence for causality [31]. Individual studies reported differences in vaginal and cervical taxa, including reduced *Lactobacillus* and enrichment of organisms commonly associated with higher-diversity communities [32]. Whether these differences persist after adjustment for obesity, insulin resistance, hormonal contraception, medication exposure, and menstrual or bleeding status is unknown because these variables have not been consistently measured or modeled. Sampling site is also critical: vaginal, cervical, endometrial, follicular-fluid, and ovarian samples are not interchangeable. Low-biomass upper-tract specimens are especially vulnerable to contamination.

Claims that *Chlamydia trachomatis* is part of a PCOS “microbiome signature” require particular caution. Detection of a pathogen, serologic evidence of prior exposure, and relative abundance in a sequencing dataset answer different questions. A positive antibody result does not demonstrate active lower-tract infection, and 16S sequencing generally lacks the validation required for clinical diagnosis.

### 5.3. Is There a Gut–Genital Microbial Axis?

The gut and genital tract share organisms and metabolic exposures, and rectal reservoirs may contribute to vaginal colonization. SCFAs can have compartment-specific effects: they support intestinal epithelial and regulatory immune functions, yet high vaginal concentrations in anaerobe-dominant communities may accompany inflammation [33]. Oral or intestinal interventions might therefore influence the vagina indirectly through immune, hormonal, or microbial routes. Conversely, genital inflammation may affect systemic immune tone.

Direct evidence for a gut–genital axis in PCOS remains insufficient. Studies should obtain paired stool and genital samples, use strain-level metagenomics to evaluate shared organisms, collect cycle and exposure data, and measure compartment-specific metabolites. Similarity at the phylum or genus level does not demonstrate translocation.

## 6. Microbial Infection Is Not Synonymous with Dysbiosis

Within the broader microbiome question, dysbiosis and infection may interact but represent different biological and evidentiary categories. Dysbiosis describes a community-level change in microbial composition or function and does not require an invasive pathogen. By contrast, active infection requires contemporaneous organism-specific evidence, such as validated nucleic-acid, antigen, culture, or clinically appropriate tissue testing; seropositivity usually indicates previous exposure and does not identify the timing, anatomic site, persistence, or current activity of infection. A cross-sectional association between an exposure marker and PCOS cannot establish temporality or causality. Nevertheless, an active or recurrent infection could theoretically modify microbial ecology, epithelial integrity, metabolites, or immune tone and thereby influence an existing PMOS/PCOS phenotype.

### 6.1. Chlamydia Species

An early nested case–control analysis from the Northern Finland Birth Cohort reported that antibodies to *C. trachomatis* and *C. pneumoniae* were more common among women with self-reported oligo-amenorrhea and hirsutism, particularly in the presence of elevated high-sensitivity C-reactive protein [34]. These serologic findings indicate previous exposure, not active infection, and the observed association did not establish infection timing, temporality, or causality. The study was hypothesis-generating, did not use contemporary PCOS phenotyping, and could not exclude behavioral or metabolic confounding. A small 2024 cross-sectional study found no statistically significant difference in *C. trachomatis* IgG between 40 women with PCOS and 40 controls [35]; this comparison likewise concerned previous exposure rather than active genital infection.

Genital *C. trachomatis* clearly causes cervicitis, pelvic inflammatory disease, tubal injury, and infertility through pathogen-specific mechanisms [36]. Those sequelae should not be recast as evidence that *C. trachomatis* causes PCOS. Experimental chlamydial infection can nevertheless serve as a defined inflammatory perturbation to test whether infection modifies a pre-existing PCOS phenotype. Such studies must distinguish ovarian function from tubal damage, pathogen burden from immune pathology, and active infection from prior exposure.

The cGAS-STING pathway is relevant to host resistance against *C. trachomatis* in the female genital tract [37]. However, the original manuscript’s proposed chain from bacterial LPS to cGAS-STING to PCOS was overly direct. LPS is canonically sensed through TLR4, whereas cGAS detects cytosolic DNA and generates cGAMP to activate STING. Cross-talk can occur, but the initiating ligand, cell type, and compartment must be specified. Evidence that genital-tract cGAS-STING activation causes PCOS is not currently available.

Although current data do not support *C. trachomatis* as a cause of PMOS/PCOS, the IL-12, type I interferon, and Th1 responses elicited by intracellular pathogens could theoretically activate STAT4 in susceptible hosts [36,37,38]. This biologically plausible connection does not imply that infection initiates PMOS/PCOS and remains a formal, entirely unproven hypothesis requiring direct testing.

### 6.2. Helicobacter pylori and Other Infections

Small serologic studies of H. pylori have produced conflicting associations with PCOS. A 2009 case–control study reported higher seropositivity [39], whereas a 2016 study found no association [40]. A 2024 cross-sectional study likewise found no significant difference in *H. pylori* or *C. trachomatis* IgG between groups [35]. In each case, antibody detection indicates previous exposure and does not demonstrate active infection, persistence, or infection preceding PCOS. Seroprevalence also varies strongly with age, geography, socioeconomic status, household exposure, and ethnicity; these small comparisons therefore cannot establish causality and are vulnerable to confounding.

Evidence involving cytomegalovirus, Epstein–Barr virus, herpes simplex virus, or varicella–zoster virus is even less specific. A population study reported an association between PCOS and increased herpes-zoster risk, but this does not establish that viral infection preceded or caused PCOS and could reflect obesity, dysglycemia, health-care use, or other comorbidities [41]. Small regional serologic reports indicate previous exposure only; they do not establish active infection, reactivation, or a PCOS-specific viral mechanism without replication and adequate control of background seroprevalence.

Integrated with the broader microbiome literature, the appropriate conclusion is that an active or recurrent infection could alter community structure, barrier function, or immune signaling and thereby modify inflammatory and metabolic phenotypes. However, evidence of previous exposure or a cross-sectional association is insufficient to demonstrate active infection or causality, and no specific infectious organism has been established as a cause of PMOS/PCOS. Antimicrobial treatment should therefore be directed by standard clinical indications, not used as microbiome therapy for PCOS. The evidence describing specific infections and their respective permissive conclusions in relation to PCOS is summarized in Table 2.

## 7. Immune Pathways Linking Microbial Signals to Ovarian Dysfunction

### 7.1. Pattern-Recognition and Inflammasome Signaling

TLR4-NF-κB signaling provides a plausible bridge between LPS exposure, cytokine production, insulin resistance, and steroidogenesis. NLRP3 inflammasome activation can process IL-1β and IL-18, while cytosolic nucleic-acid sensors—including cGAS-STING—respond to host- or microbe-derived DNA in a cell- and context-dependent manner [42,43]. Mitochondrial stress in obesity can activate cGAS-STING independently of infection [44]. Thus, the same downstream inflammatory pathways may be engaged by sterile metabolic injury and microbial exposure.

This convergence complicates causal interpretation. Elevated IL-1β, TNF, or interferon-stimulated genes do not identify the upstream trigger. Mechanistic studies should measure the relevant ligand, receptor engagement, cellular source, and response to pathway-specific inhibition. Whole-ovary cytokine measurements cannot determine whether the signal originated in theca cells, granulosa cells, macrophages, endothelial cells, or infiltrating lymphocytes.

### 7.2. T-Cell Polarization, Regulatory Restraint, and STAT4

STAT4 is activated most prominently downstream of IL-12 through JAK2 and TYK2 and is required for optimal T-helper-1 differentiation and interferon-γ production [38]. It also participates in responses to IL-23 and type I interferons in selected immune cells. In inflammatory environments, STAT4 can therefore amplify Th1-associated programs. Regulatory T cells ordinarily limit these responses. T-bet-positive regulatory T cells show delayed IL-12 receptor β2 expression, which restrains STAT4-dependent conversion toward a Th1-like state and preserves suppressive function [45]. The simple statement that STAT4 “inhibits peripheral Tregs” is therefore incomplete: the outcome depends on differentiation stage, receptor expression, tissue context, and cytokine timing.

Direct evidence linking lymphocyte STAT4 activation to human PCOS susceptibility has not been identified. Its prominence instead reflects its worthwhile investigative potential as a specific, biologically coherent, and falsifiable hypothesis connecting microbial or sterile inflammatory signals with immune polarization and ovarian dysfunction. In a susceptible subgroup, exaggerated IL-12-STAT4 responsiveness could favor Th1/IFN-γ activity, weaken local regulatory restraint, and interact with hyperandrogenism, macrophage activation, and granulosa-cell stress in the ovary. However, this is not an established disease mechanism and requires direct validation in human PMOS/PCOS.

### 7.3. Granulosa-Cell STAT4 and Sex-Steroid Modulation

In non-PCOS experimental systems, STAT4 has been reported to bind the KISS1 promoter and promote apoptosis while reducing estradiol synthesis in porcine granulosa cells [46]. Additional porcine and mouse follicle experiments linked reduced methylation of the STAT4 promoter to higher STAT4 expression, granulosa-cell apoptosis, impaired proliferation, reduced estradiol, and delayed follicular development [47]. These studies provide mechanistic insight into a possible ovarian-cell-autonomous role for STAT4, but they were conducted in porcine or murine systems, were not performed in human PCOS tissue, and therefore require direct human validation before generalization.

Estrogen can increase IL-12-induced STAT4 phosphorylation in murine T cells and preferentially activate a STAT4β isoform in mouse splenic lymphoid cells [48,49]. These findings are relevant to immune signaling but do not demonstrate that estrogen promotes PCOS through STAT4. Estrogen has dose-, receptor-, tissue-, and disease-specific immunologic effects, many of which are anti-inflammatory. Evidence that progesterone directly inhibits STAT4 in PCOS is not established. Progesterone can dampen T-cell activation and promote regulatory programs through other pathways, but its net ovarian and immune effects cannot be inferred from STAT4 alone.

Proposed testable hypothesis: The following pathway is presented as a hypothesis with worthwhile investigative potential, not as an established mechanism in human PMOS/PCOS. In a metabolically susceptible host, intestinal or genital microbial signals may increase antigen-presenting-cell IL-12, producing exaggerated STAT4 phosphorylation in effector lymphocytes. A permissive ovarian environment, shaped by hyperandrogenism, altered cyclic steroid exposure, and granulosa-cell stress, could then convert systemic Th1 bias into local follicular dysfunction. Parallel granulosa-cell STAT4 activation may increase apoptosis through KISS1-related or other pathways. The model predicts that disease severity would track with cell-specific pSTAT4 rather than total STAT4 and that lymphocyte and granulosa-cell effects could be experimentally separated.

Testing this hypothesis requires: (1) flow cytometric measurement of basal and IL-12-stimulated pSTAT4 in defined lymphocyte subsets; (2) ovarian immunostaining or single-cell transcriptomic/proteomic localization; (3) Th1, Th17, and regulatory T-cell phenotyping; (4) estrous-stage and hormone measurements; (5) cell-specific perturbation rather than systemic JAK inhibition; and (6) evidence that pathway modification changes ovarian outcomes without merely improving body weight or insulin sensitivity. The candidate microbial-immune ovarian mechanisms and their respective evidence strength are summarized in Table 3.

## 8. Animal Models and Causal Inference

Animal models are indispensable for testing directionality and provide valuable insight into potential mechanisms underlying PCOS pathophysiology. However, they reproduce selected reproductive or metabolic features rather than the full, heterogeneous human syndrome. Consequently, findings from these models should be interpreted as mechanistic or hypothesis-generating evidence and require confirmation in rigorously phenotyped human cohorts. Letrozole exposure increases androgen synthesis by inhibiting aromatase and can generate persistent estrous disruption, polyfollicular ovarian morphology, hyperandrogenism, and metabolic changes. Dihydrotestosterone models provide non-aromatizable androgen exposure, while prenatal androgen models examine developmental programming. Model age, exposure route, dose, strain, diet, housing, and treatment continuation materially change phenotype [50,51,52,53].

Microbiome research introduces additional variables. Cage, vendor, bedding, chow, water, and coprophagy can influence microbial composition as strongly as the experimental treatment. Cohousing is not equivalent to controlled fecal microbiota transfer because it exchanges microbes, metabolites, hormones, and environmental exposures. Stool does not fully represent mucosa-associated communities. Investigators should randomize cages, report litter and vendor, avoid housing all animals of one group together, and treat cage as an experimental unit where appropriate.

Several animal experiments support causality within their respective models. Healthy-donor fecal transfer or *Lactobacillus* administration improved reproductive and metabolic features in a rat PCOS model [54]. Cohousing letrozole-treated mice with controls modified microbial communities and protected against reproductive and metabolic dysfunction [55]. Transfer of microbiota from women with PCOS to mice produced ovarian and metabolic abnormalities, showing that a human-derived microbial community can transmit selected features under controlled experimental conditions [56]. The *B. vulgatus*–bile acid–IL-22 experiments added defined-organism and rescue evidence in mice [25]. Collectively, these data illuminate potential pathophysiologic mechanisms, but they do not demonstrate that FMT, particular organisms, or the bile-acid–IL-22 pathway cause or reverse PCOS in humans.

These studies do not establish FMT as a safe or effective human PCOS treatment. Instead, they provide a platform for investigating microbial contributions to disease pathophysiology under controlled experimental conditions. Translation requires confirmation of the implicated organisms, metabolites, pathways, and clinical effects in humans. A rigorous animal experiment should include disease induction, treatment while the inducing condition continues, vehicle and healthy controls, cage-balanced randomization, prespecified reproductive and metabolic outcomes, and confirmation that the intervention changes the intended microbial or barrier target. Summarized in Table 4 are the experimental models relevant to microbial mechanisms. 

## 9. Therapeutic Implications

### 9.1. Established Care Versus Microbiome-Mediated Effects

Lifestyle treatment, combined hormonal contraception, metformin, antiandrogens, anti-obesity pharmacotherapy, and fertility-directed treatment should follow evidence-based PCOS guidance [3]. Several established interventions can alter the microbiome, but a microbiome change observed after treatment does not prove that it mediated clinical benefit. Weight loss, dietary composition, improved glycemia, altered bile acids, and medication exposure can independently change both endocrine outcomes and microbial communities.

Microbiome studies embedded within treatment trials should therefore prespecify mediation analyses and collect baseline and serial stool, metabolite, diet, and medication data. A treatment should not be labeled “microbiome-directed” merely because microbial composition changes after the clinical phenotype improves.

### 9.2. Probiotics, Prebiotics, and Synbiotics

Small randomized trials and meta-analyses suggest that probiotic or synbiotic supplementation may modestly improve insulin resistance and selected lipid, inflammatory, or androgen measures [22,57,58,59,60]. Effects are inconsistent across outcomes and products. Formulations differ in species, strain, viability, dose, prebiotic substrate, duration, background diet, and adherence. Many trials are single-center, include small samples, and do not measure microbial engraftment or function. Publication bias and multiple outcome testing are additional concerns.

The six-month trial by Chudzicka-Strugała and colleagues is notable for its duration and improvements in testosterone, lipids, LPS, and LPS-binding protein when synbiotics were added to intensive lifestyle modification [22]. Yet high attrition and absence of microbiome sequencing limit mechanistic interpretation. Probiotic and synbiotic products remain investigational adjuncts rather than standard disease-modifying therapy.

### 9.3. FMT, Defined Consortia, Metabolites, and Immune Targets

FMT has not been established as safe or effective for human PCOS. Evidence of benefit and mechanistic rescue currently comes from animal models, whereas human FMT trials in PCOS are lacking. Animal findings justify further mechanistic and carefully designed translational studies but not clinical use. Whole-community transfer also creates challenges involving donor variability, transmissible pathogens, antimicrobial-resistance genes, phages, metabolites, and durability. Defined microbial consortia or postbiotics may eventually be more controllable, but they require evidence of composition, pharmacology, target engagement, and clinical benefit in humans.

The bile-acid–IL-22 and SCFA-related pathways offer tractable experimental targets [23,24,25], but neither pathway is established as a causal therapeutic target in humans with PCOS. Any intervention must demonstrate in human studies that it changes the proposed pathway and improves reproductive outcomes beyond nonspecific metabolic improvement. Systemic cytokine or JAK-STAT manipulation carries substantial immune risk. STAT4-directed experiments should begin with cell-specific mechanistic studies, not empiric anti-inflammatory treatment. Similarly, TNF inhibitors or other antirheumatic drugs should not be proposed as PCOS treatments without a compelling target, safety rationale, and disease-specific efficacy evidence.

Antibiotics have no role in treating PCOS-associated dysbiosis in the absence of a diagnosed infection. They may worsen ecological disruption, select resistance, and obscure mechanistic studies.

## 10. Research Priorities

The field is now limited less by the number of association studies than by design quality. Seven priorities would materially advance translation.

1. Deep and standardized phenotyping. Apply contemporary diagnostic criteria; report phenotype, age, race and ethnicity, adiposity, insulin sensitivity, diet, medication, menstrual or hormone-exposure status, sleep, smoking, and recent antibiotics. Avoid pooling adolescents and adults without stratification.
2. Longitudinal and transition-focused sampling. Repeated sampling should bracket puberty, weight change, treatment initiation, ovulation induction, and pregnancy. Temporal data are essential to separate microbial cause, consequence, and treatment response.
3. Compartment-specific multi-omics. Combine shotgun metagenomics, metatranscriptomics where feasible, targeted and untargeted metabolomics, validated permeability measures, immune phenotyping, and ovarian or follicular-fluid analysis. Paired stool and genital sampling should use contamination controls and absolute microbial quantification.
4. Integrative analytic frameworks. Prioritize microbial gene modules and metabolic pathways over genus-level abundance, and link shotgun metagenomic capacity to measured bile acids, SCFAs, and tryptophan metabolites and to host transcriptomic or immune signatures within the same participants. Longitudinal mediation analysis can test whether a microbial product or host pathway statistically mediates an exposure–outcome relationship, but meaningful interpretation requires correct temporal ordering and control of exposure-mediator, mediator-outcome, and time-varying confounding. Mendelian randomization may provide complementary causal evidence when valid genetic instruments are available, although weak instruments, horizontal pleiotropy, population structure, and limited reproducibility of microbiome traits require caution. Candidate mechanisms should be replicated in independent human cohorts and triangulated with perturbation and rescue in animal models and receptor- or cell-specific validation in vitro before being considered clinically actionable.
5. Reproducible analytics. Prespecify pipelines, share raw data and metadata, validate classifiers in external regions, and correct for compositionality and batch effects. Apparent high discrimination within a training dataset is not a clinical biomarker.
6. Mechanistic triangulation. Prioritize findings supported by human association, model perturbation, and rescue. Defined organisms, metabolites, receptors, and cell-specific pathways are more informative than broad community shifts alone.
7. Clinically meaningful outcomes. Trials should assess ovulation, menstrual regularity, validated hyperandrogenism outcomes, glucose tolerance, patient-reported outcomes, and fertility where appropriate—not only HOMA-IR, cytokines, or relative taxonomic abundance.

For the STAT4 hypothesis specifically, initial studies should determine whether pSTAT4 is elevated in lymphocyte subsets or ovarian cells in rigorously phenotyped human samples. A mouse study should then test temporality and cell specificity. A positive result would require pathway rescue or genetic perturbation; a simple correlation between total ovarian STAT4 and PCOS-like morphology would be insufficient.

## 11. Conclusions

Microbial ecology is likely one component of the heterogeneous PMOS/PCOS network, but current evidence does not support a single dysbiotic signature or a specific infectious cause. Human data most consistently show context-dependent differences in gut community structure, while causal support comes mainly from transfer, defined-organism, and rescue experiments in animals. These models provide important pathophysiologic insight but do not fully capture the clinical and biological heterogeneity of human PMOS/PCOS; their findings therefore require validation in longitudinal human studies before clinical translation. The lower genital tract is understudied, and gut–genital crosstalk remains a plausible but unproven construct.

The most productive next phase will replace catalogues of taxa with integrated measurements of microbial function, metabolites, epithelial integrity, immune-cell state, and ovarian biology. Infection and dysbiosis must remain conceptually distinct, and studies should separately report previous exposure, active infection, cross-sectional association, temporality, and evidence sufficient for causal inference. IL-12-STAT4 signaling is presented as a hypothesis with worthwhile investigative potential because it offers a specific and falsifiable bridge between microbial sensing, Th1 polarization, and granulosa-cell stress. However, direct human PCOS evidence is absent, however, and the pathway should not be regarded as an established mechanism unless supported by cell-specific and longitudinal human validation.

## Figures and Tables

**Table 1:** Representative human gut microbiome evidence in PCOS.

Evidence Source	Design and Principal Observation	Interpretation	Major Limitation
Lindheim et al., 2017 [12]	Pilot case–control study; altered community profile and lower diversity; no group-level barrier/endotoxemia signal	Established early human association	Small sample; cross-sectional
Liu et al., 2017 [13]	Case–control study linked dysbiosis with clinical parameters	Suggested phenotype–microbiome relationships	Geography, BMI, diet, and sequencing confounding
Torres et al., 2018 [14]	Gut diversity correlated with hyperandrogenism	Supports endocrine–microbial coupling	Correlation cannot determine direction
Yang et al., 2024 [15]	Individual-level reanalysis of 14 studies and 948 participants; no overall alpha-diversity difference; region- and testosterone-dependent taxa	Strong evidence against a universal taxonomic signature	Public datasets remain heterogeneous and largely cross-sectional
Hanna et al., 2025 [16]	Systematic review of human and animal composition, metabolites, and interventions	Functional pathways may be more informative than taxa	Marked between-study heterogeneity
Chudzicka-Strugała et al., 2025 [22]	Randomized synbiotic plus lifestyle trial; improved several endocrine/metabolic markers and reduced LPS/LBP	Preliminary human intervention signal	High attrition; no microbiome sequencing

**Table 2:** Infection-related evidence and permissible conclusions.

Organism or Exposure	Current Evidence in PCOS	Permissible Conclusion	What Would Strengthen Inference
*C. trachomatis*	One older symptom-based serologic association; a recent small study was negative [34,35]	Prior exposure may correlate with selected symptoms in some populations; causality is unproven	Prospective NAAT-confirmed infection cohorts with pre-infection endocrine phenotyping
*C. pneumoniae*	Older serologic association with self-reported PCOS-like symptoms [34]	Hypothesis-generating only	Replication with validated PCOS diagnosis, timing, and confounder adjustment
*H. pylori*	Small conflicting serologic studies [35,39,40]	No consistent association	Large, geographically diverse cohorts using active-infection testing
Herpesviruses	Sparse regional serology and administrative-data associations [41]	No established etiologic role	Background-seroprevalence controls, viral-load/reactivation measures, prospective design
Higher-diversity vaginal communities	Limited PCOS sequencing studies [31,32]	Genital community structure may differ in PCOS	Paired, longitudinal, cycle-aware and medication-aware sampling with absolute quantification

**Table 3:** Candidate microbial–immune–ovarian mechanisms and evidence strength.

Mechanism	Human Association	Causal Experimental Support	Current Assessment
Gut community alteration ↔ hyperandrogenism	Moderate; replicated at community level, taxa inconsistent [14,15]	Androgen and microbiota manipulations alter phenotype in models	Bidirectional relationship is likely; direction in humans unresolved
Barrier dysfunction/LPS → TLR4-NF-κB → insulin resistance/androgen production	Limited and mixed [12,20,21,22]	Strong general metabolic evidence; ovarian steroidogenic responses reported [19,21]	Plausible in a subset; not a universal PCOS mechanism
*B. vulgatus* → altered bile acids → reduced IL-22	Limited human replication	Transfer, knockout, and rescue evidence in mice [25]	Strong translational hypothesis requiring independent human validation
SCFAs → epithelial and Treg effects	Indirect and inconsistent	Strong general mucosal-immunology evidence [23,24]	Function and compartment must be measured directly
Vaginal dysbiosis → mucosal inflammation → ovarian dysfunction	Limited [31,32]	General genital-inflammation evidence; PCOS-specific causality lacking	Association only
Infection → chronic inflammation → PCOS	Sparse and conflicting [34,35,39,40]	No organism-specific PCOS causal model	Unproven
IL-12-STAT4 → Th1 bias and/or granulosa-cell apoptosis	No direct human PCOS evidence	Immune evidence plus porcine/mouse granulosa-cell studies [38,45,46,47,48,49]	Explicitly hypothesis-generating

**Table 4:** Experimental models relevant to microbial mechanisms.

Model or Perturbation	Strength	Key Limitation	Most Appropriate Use
Letrozole-induced mouse PCOS [51,52,53]	Reproducible reproductive phenotype; compatible with microbiome sampling	Age, dose, route, and continuation alter metabolic and microbial outcomes	Testing whether an intervention modifies an established hyperandrogenic phenotype
DHT model [50]	Stable non-aromatizable androgen exposure	Does not model all endocrine feedback; androgen directly alters microbiota	Separating androgen effects from aromatization
Healthy-donor FMT or *Lactobacillus* in PCOS rodents [54]	Direct microbial intervention	Donor variability; transfer may include metabolites and phages	Testing whether community replacement modifies phenotype
Cohousing [55]	Low-cost microbial exchange	Uncontrolled exchange of microbes, metabolites, steroids, and environment	Exploratory evidence, not a precise FMT surrogate
Human-to-mouse PCOS FMT [56]	Tests transmissibility of a human community phenotype	Recipient ecology and species differences	Prioritizing candidate functions for mechanistic follow-up
Defined *B. vulgatus*, bile-acid, and IL-22 perturbation [25]	Organism-level, pathway, knockout, and rescue evidence	Replication and human generalizability remain limited	Testing a defined microbial–metabolite–immune pathway

## Data Availability

No new data were created or analyzed in this study. Data sharing is not applicable to this article.
